# Tumour expression of leptin is associated with chemotherapy resistance and therapy-independent prognosis in gastro-oesophageal adenocarcinomas

**DOI:** 10.1038/bjc.2016.139

**Published:** 2016-05-26

**Authors:** G H Bain, E Collie-Duguid, G I Murray, F J Gilbert, A Denison, F Mckiddie, T Ahearn, I Fleming, J Leeds, P Phull, K Park, S Nanthakumaran, K M Matula, H I Grabsch, P Tan, A Welch, L Schweiger, A Dahle-Smith, G Urquhart, M Finegan, R D Petty

**Correction to:** *Corrigendum: British Journal of Cancer* (2015) **113**, 1641–1641; doi:10.1038/bjc.2015.391; published online 1 December 2015

Upon publication of the above corrigendum in the *British Journal of Cancer*, the authors noted that author KM Matula had been placed in the incorrect position on the author listing. The publishers would like to apologise for this mistake. The full and correct author listing is reproduced above.

